# Service user involvement: impact and participation: a survey of service user and staff perspectives

**DOI:** 10.1186/s12913-014-0491-7

**Published:** 2014-10-25

**Authors:** Edward Omeni, Marian Barnes, Dee MacDonald, Mike Crawford, Diana Rose

**Affiliations:** Service User Research Enterprise (SURE), Institute of Psychiatry, Psychology and Neuroscience at King’s College London, De Crespigny Park, London, SE5 8AF UK; School of Applied Social Science, University of Brighton, Mayfield House, Falmer, East Sussex BN1 9PH UK; Department of Medicine, Imperial College, Claybrook Centre, Claybrook Road, , Hammersmith London, W6 8LN UK

**Keywords:** Mental health services, Service user involvement, Patient and public involvement, Health services research

## Abstract

**Background:**

Over the last 20 years governments around the world have promoted user involvement in an effort to improve the quality of health services. Despite the growing emphasis placed on user involvement in England, there is a paucity of recent studies looking at how service users and professionals perceive the outcomes of user involvement policies. This study aimed to examine the overall levels of participation in service user involvement in mental health services among professionals and service users and ascertain their views on the impact of involvement activity on various areas of service delivery.

**Methods:**

A cross-sectional survey of service users and providers within community mental health services. The sampling was carried out across three mental health Trusts, two serving people living in inner-city areas and a third covering a mixed rural/urban population. A questionnaire with closed and open ended questions was used to gather the responses of service users and frontline professionals. As a mixed methods study, the analysis consisted of both quantitative and qualitative approaches.

**Results:**

Three hundred and two service users responded to the survey with a response rate of 48%. One hundred and forty three frontline mental health professionals, 26.8% of those approached submitted questionnaires. Almost half of service users (N=138, 45.7%,) and healthcare professionals (N=143, 55.9%) reported having been involved in some form of user involvement activity. Although there were some differences in the responses of service users and frontline professionals, both groups reported that service user involvement was having a positive impact.

**Conclusions:**

The findings show that, within the three mental health trusts examined in this study, service user involvement has become widespread and is perceived by both staff and service users to be a good policy. The study had some important limitations. The questionnaire used was based on existing literature, however it was not subjected to psychometric testing. In addition, response rates were low, particularly among professionals. Despite the limitations, the findings are encouraging, offering important of insight into views and experiences of service users and healthcare staff. Further studies are needed to assess and investigate the topic on a national level.

## Background

In the last three decades governments across Europe and North America have placed increased emphasis on service user involvement and its role in the planning and delivery of healthcare services. User involvement has been promoted by the World Health Organisation and several countries have developed legislation strengthening the influence of service users and giving them greater control over the services they receive [1–4]. This has been especially true in mental health.

A number of studies have highlighted the benefits of user involvement. It has been credited for improving the information and accessibility of services [5]. Improvements have also been observed in the coordination of care and in the relationships between clinicians and those receiving treatment [6–9]. User involvement has also been associated with positive clinical outcomes, such as improved self esteem and confidence, as well as therapeutic benefits resulting from increased social interaction [10].

Despite this rapid increase in awareness, service user involvement has struggled to overcome significant challenges associated with translating the rhetoric of empowerment and participation into practice [11].

Several studies have examined how user involvement is conducted in health services [12–16]. Research has shown that service users have found it difficult to influence service providers and have a real impact on decision-making across all levels of service delivery. Kent and Read [12] suggested that service user involvement may be progressing faster at the level of individual treatment than at a wider organisational level. Similar findings were made by Storm et al. [13], who studied service provider perspectives on service user involvement in the Norwegian context. The authors surveyed 184 service providers’ examining reports of user involvement at the individual and departmental levels of community mental health centres. They concluded that service user involvement was occurring on an individual level and service users were involved in decisions about their own treatment; however, there was still considerable progress to be made in involving service users at a departmental level.

Other studies have highlighted issues, such as staff and organisational resistance, as significant barriers to effective user involvement [2,17–19]. In a study evaluating the outcomes of a service user involvement initiatives, Storm et al. [20] suggests that service user involvement initiatives may not always translate into perceived improvements to services and increased satisfaction with care. Similarly, service improvement initiatives designed to increase awareness of user involvement and enhance participation are not always effective in influencing professional knowledge, practice or attitudes towards user involvement [21]. Researchers and activists have warned against the dangers of ‘tokenism’ and service users frequently reported limited if any benefit from their involvement in services [2,22]. In a 2002 UK based study, carried out by Rutter et al. [23], only 6 out of 25 representatives of service user groups were satisfied with the outcomes of their participation in involvement activity.

Regardless of these challenges, in the last 20 years, UK mental health Policy has continued to promote service user involvement. Successive governments have emphasised the involvement of service users as a means of increasing the acceptability and quality of services [24–26]. The 1990 NHS and Community Care Act established formal requirements for service user involvement in the planning of services. The New Labour government continued these developments with the 1999 National Service Framework for Mental Health [4] which positioned service user involvement as one of its central tenets. Developments such as the 2001 Social Care Act, further consolidated the increased focus on user involvement by setting out requirements for all NHS organisations to ensure active participation in treatment decision-making, as well as the planning and evaluation of services. In recent years the coalition government has continued to make changes in the structure of the NHS, emphasising strategies which may give people more choice and control over how their support needs are met [27].

Despite the rapid growth and mainstreaming of user involvement in recent years, the impact of these policies on the experiences and perceptions of mental health service users and providers has rarely been examined. Given this relative paucity of recent empirical research it is important to understand how the concept of user involvement is perceived by service users and frontline mental health professionals.

### Study aim

This study examines the overall levels of participation in service user involvement across three mental health trusts in the UK. The study also explores the views of service users and professionals on the impact of service user involvement on various areas of service delivery. The main research questions were:

1. What are the overall levels of participation in service user involvement initiatives among service users and frontline professionals (social workers and psychiatric nurses)?
2. What are the perceptions of service users and mental health professionals on the impact of service user involvement on key areas of service planning and delivery?
3. What positive and negative aspects do service users and mental health professionals associate with service user involvement?

In addition, the following hypotheses were examined:

- Social Workers are more likely to participate in user involvement and associate benefits with user involvement than psychiatric nurses
- High levels of service user involvement are more difficult to achieve in mostly rural, compared to mostly urban areas.

## Methods

We conducted a cross-sectional survey of service users and professionals within community mental health services. The survey was carried out in three mental health trusts covering a combined area of over 4.5 million residents, of whom around 220,000 are in contact with mental health services. The two inner city Trusts (A and B) service a younger and more ethnically diverse population with greater mental health needs than in other parts of England [28]. Trust C covered a larger, predominantly rural area. The characteristics of each of the study locations are summarised in Table 1.

**Table 1:** **Summary characteristics of the three study sites covered in the study**

**Trust**	**Population Coverage**	**Service User Population**	**Staff Employed**	**Service Sites**	**Setting**
A	1 100 000	50000	4800	100	metropolitan
B	2 500 000	70000	7000	150	metropolitan
C	1 500 000	100000	5000	120	mixed rural/urban
Total	5 100 000	220 000	16800	370	

The sampling of the three trusts was conceptual with all study locations selected on the basis of their characteristics. Both collective and individual forms of involvement were of interest, as well as the impact of factors, such as organisational change and service reorganisation, topical concerns considering the significant changes in the structure of the NHS implemented by the UK coalition government since 2010. Trust A had been undergoing significant service reorganisation. Trust B had also undergone recent restructuring and had begun placing significant emphasis on service user representation on the Trust Board. A third location (Trust C) , was added to include the perspectives of service users and staff living in rural areas, as rurality may impact on the nature and outcomes of user involvement. Trust C encompassed an area of about 1,500 square miles including 2 county councils, one city council and three separate social services authorities. The rural setting and size create a number of logistical problems, such as overcoming the difficulty of geographic dispersal and creating opportunities for service users to meet amongst themselves and trust officials and third sector providers.

Based on data obtained from trust managers, all three organisations held similar strategic approaches to user involvement. Structures of service user involvement were present at individual, service and organizational level. Each trust had service user representatives on their boards. The numbers of governors in the three trusts studied were:

- Trust A: 39 total, 26 elected (of whom 9 are Service Users), 13 appointed.
- Trust B: 36 total, 25 elected (of whom 7 are Service Users), 11 appointed
- Trust C: 41 total, 27 elected (of whom 12 are Service Users), 14 appointed.

In addition, all of the trusts continued to commission a small number of user led organizations for the purposes of consultancy, monitoring and providing additional services such as vocational courses, advocacy and peer support.

### Data collection

Fieldwork began in July 2011 and was completed in April 2012. A questionnaire with closed and open ended questions was used to collect the responses of both service users and frontline professionals. Professionals were invited to participate using a self-completion online questionnaire, while service users were approached directly by a member of the research team. A different method was adopted for both groups as we aimed to include a wide range of service users including those who may not have had regular internet access.

The survey addressed the respondents’ experience of participating in user involvement initiatives as well as their views about the impact of various forms of user involvement activity. The data collection research team consisted of 5 members including 3 research assistants from the Mental Health Research Network. Survey interviews with service users were carried out on a 1 to 1 basis. The duration of each interview was between 10 to 20 minutes. During the initial phase of the interview respondents were screened for eligibility and provided with information about the study. Verbal consent was sought before proceeding with the survey questions. Considerable care was taken to avoid exerting pressure when eliciting responses from the survey participants. Participants were assured about the anonymity of their information and were encouraged to express themselves freely and independently when completing the questionnaire. Some respondents preferred to give verbal responses to the open ended questions of the survey. In order to minimise the possibility of misinterpretation, the researchers transcribed the responses verbatim, and gave the participant the opportunity to review the transcribed text and request changes if necessary.

Professionals were contacted by email one week prior to the distribution of the electronic survey, giving them the opportunity to review the informational material and opt out of receiving the electronic questionnaire. Basic demographic information was collected but no respondent could be identified.

### Sample

We aimed to collect responses from 100 service users and 42 frontline professionals (21 Community Psychiatric Nurses and 21 Social Workers) per Trust.

Broad criteria for the selection of service users were applied. These consisted of the following: (a) People above the age 18 years and (b) attending community mental health services for the purpose of treatment and/or assessment. Service users in inpatient settings were not included in the sampling for this study. A purposive sampling method was used and service user participants were recruited from community mental health clinics and local day centres and community based substance misuse services. Quotas were not applied for the recruitment distributions across age groups, gender, ethnicity or diagnosis. It was hoped that by identifying service users through ordinary clinical settings, such as waiting rooms in outpatient clinics it would be possible to obtain the views of a wide range of people attending services in the sampled trusts.

The sampling frame for the survey of frontline mental health professionals was drawn up using data from the human resources departments of each Trust. The survey was targeted at social workers and psychiatric nurses, the main professional groups in community mental health services in England. We were interested in their views, seeing them as relevant informants considering their day-to-day involvement with service users and participation in a wide range of activities including therapeutic work, care planning and management. (The survey was part of a wider research project which included in depth interviews with psychiatrists, senior clinicians, managers and commissioners. These findings will be reported elsewhere.)

Emails were sent to a random sample of psychiatric nurses and social workers asking them to complete the on-line survey. Due to the lower than expected response rate a number of Social Workers and Psychiatric Nurses were approached in person and asked to complete a paper version of the questionnaire. This was based the follow up recruitment of professionals who reported not being able to access the questionnaire electronically due to firewall issues affecting a small number of computers in Trust A and B (n= 41). A further 7 participants were recruited following visits to mental health clinics, which had not been covered in the original sampling for the study.

We predicted that social workers would be more likely than community psychiatric nurses to (1) have participated in service user involvement initiatives and (2) associate a positive benefit with such activity. There is support in the literature for this [12] and user involvement is a mandatory part of social work education. The sample size was based on the ability to test the hypothesis of a difference in mean outcome scores between two independent groups; social workers and community psychiatric nurses. We therefore estimate the sample size to be able to detect a standardised effect size of 0.5, considered a medium effect size. To be able to detect at least this magnitude of a difference with 80% power at the 5% level of significance (2-sided) we needed 63 participants in each group and this corresponds to 21 in each group per Trust as given above.

### Questionnaire design

The survey questionnaire was based on a core set of questions derived from reviews of the literature conducted by Rose et al. [29] and Crawford et al. [5]. The survey design also built on the findings generated from the Rose et al’s [30] user led study investigating the perceptions of activist and non-activist service users on the outcomes of user involvement.

The questionnaire consisted of 3 parts.

Section 1 of the questionnaire contained a series of examples of user involvement activities and service users were asked to identify which, if any, forms of involvement they had participated in. Participants were asked about their involvement in the following areas.

1. *Involvement in running day services*
2. *Involvement in running residential services*
3. *Involvement in changing in-patient wards*
4. *Involvement in appointing staff*
5. *Involvement in training staff*
6. *Involvement in managing services*
7. *Involvement in evaluating services*
8. *Involvement in researching services*
9. *Involvement in commissioning services*

They were also given the option of stating “non involvement” in any of the areas of service user involvement activity, as well as, a free text box to identify other areas of service user involvement they may have experienced.

Based on a modified template of the service user questionnaire Section 1 of the survey targeting professional included the same list of user involvement activities. Instead of personal participation, professionals were asked to identify areas where they had direct experience of involving service users.

Section two of the questionnaire measured service user and staff perceptions about the impact of service user involvement within different contexts of mental health service delivery. Using a five point Likert scale both professional and service user participants could rate the impact as: 1. strongly positive, 2 slightly positive 3 (Having) No Impact, 4 Slightly negative, 5 Strongly negative. Participants were also given the option of answering “I Don’t Know” to any of the questions. The questions focused on the 9 areas of service user involvement listed in Section one of the questionnaire. In addition participants were asked about the overall impact of user involvement. The questions in section two of the questionnaire were presented as follows:

1. *What impact have users had when they have been involved in day services?*
2. *What impact have users had when they have been involved in residential services?*
3. *What impact have users had when trying to make changes on inpatient wards?*
4. *What impact have users had when they have been involved in appointing staff?*

Section two of the questionnaire also included a series of open-ended questions. Participants were asked to identify the positive and negative impact of user involvement activity. Those who were unwilling or unable to write their responses in the open ended text box sections of the questionnaire could give a verbal response. In such cases, answers were transcribed by the interviewing researcher.

In Section three of the questionnaire service user participants were asked to provide additional details, including their age (in age bands), ethnicity and gender. Service users were asked additional information about their diagnosis and length of time they had been in contact with mental health services. Mental health professionals were also asked about their professional background (social work, nursing) and length of employment within mental health services.

### Data analysis

The analysis of quantitative data involved calculating the frequency and distribution of survey responses. Descriptive statistics were used to: (1) examine the extent of participation in different types of user involvement activities and (2) determine the total proportion of service users and staff who felt that involvement was having a positive impact. All quantitative data analysis was conducted using SPSS (version 20). We used a binary logistic regression to examine factors associated with whether or not service users and front-line professionals had been involved in user involvement activities. Diagnostic categories were omitted from the list of predictor variables due to the low response rate associated with this question in the survey. The dichotomous dependent variable was calculated as the response given by professionals and service users, to the question of whether or not they have had been involved in user involvement activity (yes/no). We used the “Enter” method to perform a standard regression analysis in which the relationship between explanatory variables and the main outcome is adjusted for the impact of all other variables in the model.

A thematic content analysis was used in the review of the data from the open ended sections of the questionnaire [31]. The analysis was inductive, although it drew on what is already known about the positive and negative outcomes of service user involvement [5–10]. Responses were read several times by the primary researchers to identify codes and themes. The overarching focus was to examine the positive and negative aspects of service user involvement and encapsulate the responses of study participants to facilitate further analysis. Coding was completed using the qualitative data analysis programme NVIVO [32].

### Ethics

The study was given ethical approval by the National Research Ethics Service Committee London (Bentham number 11/LO/0584). Agreement was sought with each participating Trust to conduct the survey. Detailed information was given to each participant about the study and its purpose. In the case of professionals the information was provided in written form due to the online recruitment strategy. In the case of service users, verbal information was provided together with supplementary information sheet. Participation in the study was voluntary and respondents could withdraw from completing the questionnaire at any time.

## Results

### Participants

Three hundred and two service users agreed to participate in the survey. Most participants (n=201, 66.6%) were recruited from community mental health and recovery clinics, with 86 (28.5%) recruited from day centres and 14 (4.6%) from community based substance misuse services. Ninety five professionals submitted a completed online questionnaire. A further 48 members of staff were approached directly by a member of the research team and completed a paper version of the questionnaire. The total recruitment figures including the response rates within each of the sampled Trusts are presented in Table 2. The descriptive statistics of our sample of service users and professionals are listed in Table 3.

**Table 2:** **Total recruitment and survey response rates by Trust**

	**Service Users**			
	**Trust A**	**Trust B**	**Trust C**	**Total**
Questionnaires distributed (n)	218	203	209	630
Questionnaires received (n)	101	100	101	302
Response rate (%)	46.3	49.3	48.3	47.9
	**Professionals**			
	**Trust A**	**Trust B**	**Trust C**	**Total**
Questionnaires distributed (n)	144	187	171	502
Questionnaires received (n)	46	49	48	143
Response rate (%)	31.9	26.2	28.1	28.5

**Table 3:** **Descriptive statistics of survey respondents**

		**Service Users (N= 302)**		**Professionals (N= 143)**	
		**n**	**%**	**n**	**%**
Age	34 or less	71	23.5	21	14.7
	35 to 49	136	45.0	82	57.3
	50 and over	95	31.5	40	28.0
Gender	Female	125	41.4	94	65.7
	Male	177	58.6	49	34.3
Ethnicity	White	193	63.9	105	73.4
	BME	109	36.1	38	26.6
Time in contact or working with services	0 to 5 years	79	26.2	13	9.1
	6 to 10 years	78	25.8	43	30.1
	Over 10 years	145	48.0	87	60.8
Professional Group	Social Worker	Not applicable	Not applicable	71	49.7
	CPN			72	50.03

### Levels of involvement and reported impact

Of the 302 surveyed service users 138, (45.7%) reported having been involved in some form of user involvement work. Participation levels among professionals were similarly high with 55.9% of the 143 mental health professionals reporting experience in at least one form of user engagement activity. The number and percentage of service users and mental health professionals involved in one or more areas of user involvement are presented in Table 4. Table 5 shows the proportion of patients and staff who had been involved in specific activities and had reported a positive impact of the area of user involvement they had experienced.

**Table 4:** **Percentage of service users and mental health professionals involved in one or more area of service user involvement**

	**Service Users**							
	Trust A		Trust B		Trust C		Total	
	n	%	n	%	n	%	n	%
Not involved	38	37.6	53	53.0	73	72.3	164	54.3
Involved	63	62.4	47	47.0	28	27.7	138	45.7
	**Professionals**							
	Trust A		Trust B		Trust C		Total	
	n	%	n	%	n	%	n	%
Not involved	18	39.1	30	61.2	15	31.3	63	44.1
Involved	28	60.9	19	38.8	33	68.8	80	55.9

**Table 5:** **Areas of involvement and reports of positive impact**

**Area of involvement**	**Service users**				**Staff**			
	**Number involved**	**% involved**	**Number reporting positive impact**	% Reporting Positive Impact*	**Number involved**	**% involved**	**Number reporting positive impact**	% Reporting Positive Impact*
Day Services	59	42.8%	51	86.4%	9	11.3%	8	88.9%
Residential services	16	11.6%	15	93.8%	4	5.0%	2	50.0%
Inpatient wards	25	18.1%	19	76.0%	13	16.3%	9	69.2%
Recruitment	18	13.0%	14	77.8%	27	33.8%	23	85.2%
Training staff	21	15.2%	16	76.2%	41	51.3%	38	92.7%
Managing services services	16	11.6%	13	81.3%	4	5.0%	3	75.0%
Evaluating services services	64	46.4%	41	64.1%	43	55.0%	37	86.0%
Research services	22	15.9%	15	68.2%	13	16.3%	12	92.3%
Commissioning services	10	7.2%	7	70.0%	5	6.3%	1	20.0%

*Denominator for calculating these percentages is the number who reported being involved in this type of user involvement activity.

**Figure 1:**
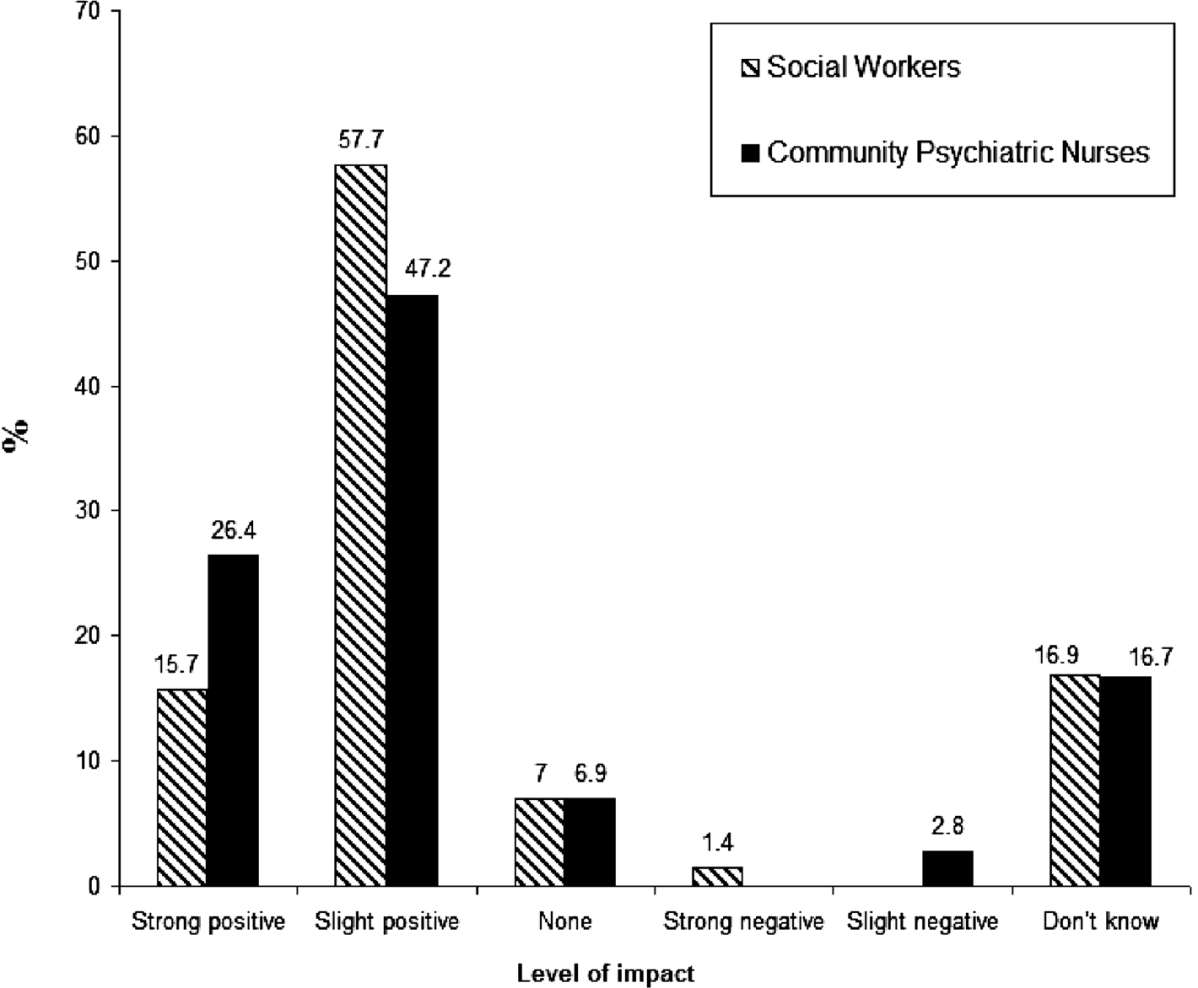
**Professional perceptions about the overall impact of service user involvement on mental health services.** *Results are broken down by professional group.

Both social workers and community psychiatric nurses associated positive benefits with service user involvement in mental health services. Over 70% in both groups felt that user involvement was having a strong or slight positive impact. In contrast, a small minority of professionals felt that user involvement was a negative or no influence. Professional perceptions about the overall impact of service user involvement on mental health services are presented in Figure 1.

Levels of reported positive impact of user involvement differed across the three trusts. Overall, 70% of service user participants in Trust A and 70.2% in Trust B reported an overall positive impact of user involvement compared to participants in the non metropolitan trust (Trust C), of whom only 51.9% felt that user involvement was having a positive impact. Levels of participation across the various areas of user involvement were also significantly lower in Trust C.

### Predictors of service user involvement

The results of the logistic regression examining factors which predict professional and service user participation in user involvement are shown in Table 6. The model shows the professionals in the age group ‘34 and under’ are more likely than other age groups to be involved in service user involvement. White professionals were associated with a higher likelihood of being involved in user involvement activity. In terms of professional background, social work professionals were more likely to be involved than community psychiatric nurses. Increasing length of employment was a further predictor associated with service user involvement. Among service user participants, increasing length of contact with services and gender were associated with a higher likelihood of participating in involvement initiatives, with men being more likely to have experienced user involvement activities than women. In terms of ethnicity, service users from BME backgrounds were more likely to be involved user involvement initiatives than white service users.

**Table 6:** **Odds ratios associated with predictors of being involved in service user involvement**

**Service Users**				**Professionals**			
**Characteristic**	**Odds ratio**	**95% Confidence interval**		**Characteristic**	**Odds ratio**	**95% Confidence interval**	
		**lower**	**upper**			**lower**	**upper**
Age				Age			
34 and under	1.00			34 and under	1.00		
35-49	0.82	0.43	1.56	35-49	0.20	0.05	0.79
50-64	0.78	0.37	1.62	50-64	0.13	0.03	0.65
Gender				Gender			
Female:	1.00			Female:	1.00		
Male:	1.26	0.74	2.13	Male:	0.92	0.41	2.08
Ethnicity				Ethnicity			
White	1.00			White	1.00		
BME	1.41	0.80	2.46	BME	0.80	0.34	1.85
Time in contact				Time employed MH			
0 to 5 years	1.00			0 to 5 years	1.00		
6 to 10 years	1.66	0.84	3.28	6 to 10 years	3.12	0.64	15.23
Over 10 years	1.97	1.03	3.76	Over 10 years	9.24	1.68	50.63
Professional Group				Professional Group			
Psychiatric Nurse	na	na	na	Psychiatric Nurse	1.00		
Social Worker				Social Worker	2.12	1.02	4.42
Trust				Trust			
Trust A	1.00			Trust A	1.00		
Trust B	0.57	0.32	1.03	Trust B	0.42	0.17	1.02
Trust C	0.27	0.14	0.53	Trust C	1.49	0.59	3.79

Service users in Trust A were associated with a higher likelihood of being involved in user involvement activity, while service users in Trust C were least likely. As for professionals, participants in Trust C were the most likely to be involved and professionals in Trust B the least likely.

### Benefits and drawbacks of service user involvement reported by service users and staff

Table 7 shows the advantages and disadvantages of service user involvement as reported by service users and staff. The results are derived form the qualitative analysis of responses to the open ended survey questions, which elicited people’s insights on the positive and negative aspects of user involvement. Ninety seven (32.1%) service users and 67 (46.9%) mental health professionals provided written or verbal comments in response to these questions.

**Table 7:** **Benefits and disadvantages of service user involvement reported by service users and staff**

**Benefits of Service User Involvement**			
Service Users	N	Professionals	
Having a say, Included in decision making	35	Service users having a say, Empowerment	26
Improvement in services	25	Therapeutic benefit, Self esteem, recovery	16
Feeling listened to, chance to give opinion	20	Improvements in services	15
Therapeutic benefit, Self esteem, recovery	13	Service user feel listened to and valued	8
Opportunities for social interaction	11	Service users a source of knowledge	7
Access to information about services	9	Access to information about treatment	3
Getting involved in groups/activities	7	Service users’ professional development	3
Improving relationship with clinicians	7	Positive use of time,	3
Opportunity to develop skills	5	Other	4
Other	4		
**Disadvantages of Service User Involvement**			
Tokenism, No resulting change	11	Service users too negative/too critical	12
Users unable to participate due to health	8	Involvement detrimental to mental health	9
User input not seen as important	6	Involved service users not representative	8
Involvement detrimental to mental health	5	User input not seen as important	6
Other	3	Tokenism, No resulting change	6
		Unrealistic demands made by service users	4
		Other	2

### Advantages of service user involvement

Overall, 35 service users and 26 professionals identified user involvement in decision making as the important outcome and this was the response most frequently highlighted as a positive benefit of user involvement. Professionals seemed to frame this particular benefit in terms of service user ‘empowerment’. Unlike the professional respondents, references to ‘empowerment’ were rarely made in the answers given by service users. Instead service users frequently articulated the ability to ‘exercise control’ and ‘choice’.

A number of service users and professionals (n= 25; n= 15) described improvement to services as a significant positive outcome of user involvement initiatives. Professional respondents often referred to user involvement as a way of making services more responsive to service user needs. Service users seemed to place less emphasis on this highlighting general service improvement and positive changes to the way service are delivered. Both professional and service user respondents (n= 16, n= 13) identified therapeutic benefits associated with user involvement, as well as the positive impact of engagement activity on self-esteem and overall recovery as a positive benefit of user involvement. In their comments on the benefits of user involvement 11 service users mentioned opportunities for social interaction as a positive aspect of user involvement. Seven mental health professionals identified service users as a valuable source of knowledge, seeing this input as a positive aspect of user involvement.

### Negative impact of service user involvement

Both service user and professional respondents generated fewer ideas about the negative impact of user involvement. Service users most frequently referred to tokenism and failure of involvement initiatives to influence change as the main disadvantage of user involvement initiatives (n= 11). In their comments on the negative impacts of user involvement, 8 service users observed that mental health issues may prevent people from taking part in user engagement activity. A number of service users (n= 6) commented on the relatively low status of service user input within mental health service. Service users also highlighted the negative impact of user involvement on the health of those of those who become involved (n= 5). Professionals highlighted the issue of overly negative and unconstructive criticism from campaigners as a negative outcome of user engagement initiatives, with 12 respondents identifying this as a problem. Issues of representativeness were also raised as a negative outcome, with 8 professionals stating that those who become involved may not be representative of the larger population of service users, thus making them unsuitable to speak on behalf of others. In their comments professionals questioned the rationale of identifying service users to represent a wider population of service users particularly if they are ‘currently well’ articulate and from a background that doesn’t reflect the views of the majority of people receiving services.

Both service users and professionals (n= 9) suggested that user involvement may negatively impact the health and self esteem of those who become involved, citing stress and the high demands associated with user involvement work as a primary cause for this. Tokenistic practices and involvement initiatives that do not lead to change were identified by 6 professionals as a significant negative aspect of service user involvement.

## Discussion

The main purpose of the study was to examine the overall levels of participation in service user involvement across three mental health trusts in the UK. In addition, the study aimed to ascertain the views of service users and professionals on the impact of user involvement on different areas of service delivery. High levels of service user involvement were observed among both professional and service user respondents. Participants who had taken part in user involvement work were likely to report a positive impact of the type of user involvement activity they had experienced. With a significant proportion of the sample recruited from community day centres, service user respondents were most likely to have participated in running day services also reporting a positive impact of user involvement in this area. Service users were also likely to have participated in service evaluation and providing feedback about mental health services. As highlighted by Beresford [33] in 2002 service user involvement in evaluation was becoming “significant and widespread” within health and social care, with funding providers and service commissioners emphasising the need for evidence that includes service user perspectives.

We found that mental health workers were most likely to have direct experience of user involvement in training, a finding which is reflected in other studies highlighting the mainstream position of user involvement in professional education and training [34–37]. In addition, a significant majority of professionals felt that service user involvement in training was having a positive impact. Fewer service users had direct experience of user involvement in training. Service users were also less likely than professionals to state that user involvement was having a positive impact in this area. The finding is interesting as it indicates that user involvement in training is highly valued by frontline clinicians with a significant number of professionals having directly experienced user involvement in this area and reporting a positive outcome of such activity. Professionals may be more aware than service users about the positive impact of users on their training and professional education, as service users may not directly see the outcome of their involvement in this area.

A significant number of both professionals and service users had experience in user involvement activity associated with service evaluation, although professionals were more likely than service users to report a positive impact of this form of engagement. This is not surprising as the results of evaluation initiatives, such as the national patient survey, are rarely fed back to patients and professionals may be more aware of the outcomes of service improvement initiatives. Both service users and professionals were least likely to have experience in the area of commissioning services. This may be due to the highly specialised nature of commissioning processes within mental healthcare settings, but it also may reflect the limited opportunities to become involved in his area of user engagement. This is explained further by the findings reported by Storm et al. [13] who concluded that involvement practices may be evolving faster on the level of individual treatment, as opposed to involvement at a departmental level where considerable progress needs to be made.

Trnobranski [38] points out that characteristics such as cultural background, age, gender and previous health care experience may influence the extent to which service users are willing to be involved in decisions about their care [39]. Organisational and professional culture, as well as the approach taken to involving service users may also determine the extent to which various groups of service users can become involved in decision making.

Based on the results of the logistic regression analysis (see Table 6) service users who had a longer history of involvement in mental health services were more likely to have experience of user involvement activity. In terms of gender, male service users were more likely to have had experience of service user involvement than female service users. The findings presented here highlight the need for further research focusing on how the approach to gender in mental health service organisation and delivery may influence participation in service user involvement. Ethnicity was also shown to be a predictor for user involvement, with service users from BME backgrounds more likely to have experience in service user involvement activity. This finding is interesting, particularly when considering the context of ongoing concerns about mental health inequalities among minority ethnic groups in England. The results may highlight the increased momentum gained by Black and Minority Ethnic service user-led groups in the two inner city Trusts covered in the study and the growing emphasis placed on involving service user from BME groups [40].

There was partial support for the first hypothesis of the study. Both groups of professionals were highly positive about service user involvement, however social workers were more likely to have direct experience of user involvement activity (see Table 6). In support of our second hypothesis we found that service users in Trust C, a mainly rural location, were less likely to participate in user involvement. When compared to Trust A and B, participation was higher among professionals, however, significantly lower among service users. In addition, only 52% of participants in Trust C felt that user involvement was having a positive impact. Factors, such as the geographical location, transport and the size of the service, may significantly determine how user engagement is experienced by service users and the extent to which they can become involved. This finding emphasises the importance of avoiding a ‘one size fits all’ approach when implementing user involvement policy and taking account of the environmental characteristics and challenges which may enhance or impede opportunities for involvement.

Similarities were found in the perspectives of users and professionals on the benefits and disadvantages of user involvement activities. Both service users and staff identified positive outcomes of user involvement, such as giving service user a say over how mental health service are delivered. Service users and professionals also highlighted improvements in services as a favourable outcome of involvement activity. Key differences were also identified. While service users identified opportunities for social interaction as a benefit of user involvement, this outcome was not mentioned by professional respondents. The finding is interesting as it underlines a key difference in perspective on the role and advantages of user involvement. As highlighted by Lindow [9] service providers may have very different priorities on a variety of aspects of service provision. While providers may perceive user involvement as being part of an overall strategy in delivering better and more responsive services, service users may derive personal benefits which are life enhancing in general.

A minority of participants highlighted negative aspects of user involvement. Amongst the disadvantages, professionals highlighted negativity and excessive criticism from service users. The ‘unrepresentativeness’ of individual service users who engage on behalf of other service users was also mentioned as a negative aspect. Rose et al. [41] cites this is a common criticism directed at user involvement. Service users who participate in user involvement activities are often labelled as unrepresentative of ‘ordinary service users’ particularly if involvement is occurring at higher levels, such as participation in strategic or departmental decision making. On the other hand, service users perceived as lacking the skills to participate at higher levels are easily overlooked for being unprofessional or misinformed.

Both service user and professionals highlighted the potential for service user involvement to harm service users. Future research should examine the negative effects of service user involvement on the health and wellbeing of those who take part, in an effort to understand how such problems arise and how they might be prevented in the future.

There is a paucity of recent research assessing the outcomes of user involvement in the UK, in particular following the more recent changes in healthcare, which have further emphasised the central importance of service user involvement in mental health service provision. The findings in this study indicate that user involvement has become widespread and mainstream across the three sampled trusts. Both service users and professionals were satisfied with the outcomes of their participation in user involvement activity. Perceptions and judgements about the impact of user involvement are largely positive, which may indicate that user involvement is perceived by both, service users and professionals, to be a good policy within mental healthcare, worthy of ongoing support and participation.

The findings provide some reason for optimism, particularly when considering the growing emphasis on user involvement across Europe and North America in recent decades. While the growing extent to which service users can exercise control remains encouraging, past studies have warned against the dangers of ‘tokenism’ highlighting staff and organisational resistance as potential barriers to meaningful involvement and lower levels of participation and awareness of service user involvement at senior organisational levels [2,13,17,22]. Literature suggests that the involvement of service users at higher decision making levels and the development of user-controlled services are have had a longer history in the USA and Canada [42,43]. While the findings presented in this study point to the mainstreaming and widespread prevalence of service user involvement, it would be prudent to learn from the American and Canadian experience and continue to expand the opportunities through which people can influence and shape the services they receive.

### Strengths and limitations

An important strength of the current study lies in its broad perspectives on service user involvement and the diverse range of user involvement mechanisms covered in the study. The design of the questionnaire allowed for a measurement of the level of participation in user involvement, as well as an overview of people’s perceptions on the extent to which various initiatives were having a positive impact. Furthermore participants were able to elaborate on their positive and negative experiences associated with user involvement. The inclusion of professionals, including social work and psychiatric nursing practitioners, added further depth to the study by encompassing multiple viewpoints on the subject, ensuring greater confidence in conclusions drawn from each group of respondents. A further strength of the study was the sampling method. Service users were recruited from ordinary clinical contexts including community mental health clinics, day centres and community based substance misuse services. All participants were recruited and interviewed in person by a member of the research team.

The survey has some important limitations. While the questionnaire we used was based on existing literature, was acceptable to service users and had strong face validity, it was not subjected to formal psychometric testing.

Although service users were asked about their experience of service user involvement in the various areas of user involvement activity, the survey did not address the level of experience of service users. There was a high degree of variation in the interpretation given by service users about the areas of user involvement covered in the survey. For example, many although not all of those who reported experience of user involvement in running day services had not participated in the management of high level decision making within such services but had contributed to in other ways, such as volunteering or had participated in organising groups and activities. Similarly, many of those who reported being involved in service evaluation had simply filled in a questionnaire or submitted a feedback form. However, regardless of the level of service user involvement, service users were optimistic about the positive impact of their engagement in user involvement activity.

Another limitation of the study is possible response bias. Despite researchers asking people to express their thoughts freely we cannot rule out the possibility that some staff and patients felt obliged to give a positive account of any experience of service user involvement activity.

People with an interest in service user involvement may have been both more likely to participate and more likely to express positive views than those with limited experience or interest in the subject. Despite these possible limitations, efforts were made by the research team to include comments (both positive and negative) from those who had not been involved in service user engagement activities.

The poor response rate to the online survey targeting mental health professionals was further limitation. Having only received responses from a small minority of professionals approached to take part in the study, we are uncertain about the extent to which these views can help us understand levels of participation and the views of providers on user involvement. Nonetheless, the responses we obtained regarding the advantages and disadvantages of user involvement offer some insight on the experiences of front line staff working in NHS mental health services.

The study was part of a larger multi-centre investigation. When we drilled down from the survey findings using qualitative, ethnographic methods, the picture was considerably more complex. These findings will be reported elsewhere.

## Conclusion

This paper set out to determine the overall levels of service user involvement among professionals and service users within three mental health trusts. The study also examined the views of service users and health care staff on the impact of user involvement on various areas of service delivery. The findings have shown that there is a high level of participation in service user involvement activity and a general endorsement that involvement has a positive impact. The percentage of service users and professionals who reported positive outcomes from activities they have been involved with was high, regardless of the type of activity they had experienced and their level of engagement in user involvement work. The findings suggest that service user involvement has become a mainstream policy across the three trusts examined in the study. Further studies are needed to assess the levels of participation and perceptions of service user involvement on a national level.
